# Blind nasoenteric tube insertion using a pharmaco-mechanical synergy protocol in frail older adults with chronic wounds: a retrospective study

**DOI:** 10.3389/fmed.2026.1743688

**Published:** 2026-06-24

**Authors:** Doudou Zhang, Qiuping Zhang, Xiling Xiao, Hongmin Luo, Huining Bian, Wen Lai, Pingyun Chen

**Affiliations:** Department of Burns and Wound Repair Surgery, Guangdong Provincial People’s Hospital (Guangdong Academy of Medical Sciences), Southern Medical University, Guangzhou, Guangdong, China

**Keywords:** chronic wounds, enteral nutrition, frailty syndrome, learning curve, nasoenteric tube, nursing, older adults, pharmaco-mechanical synergy protocol

## Abstract

**Objective:**

This study aimed to evaluate the feasibility and preliminary outcomes of a pharmaco-mechanical synergy protocol for blind nasoenteric tube (NET) insertion in frail older adults with chronic wounds.

**Methods:**

We conducted a retrospective analysis of 21 older chronic wound patients who underwent the protocol-guided blind NET insertion in our Wound and Ulcer Repair Department between October 20, 2021, and May 16, 2025. We assessed procedural success rates, complication rates, nutritional markers, and infection indicators, comparing pre-insertion baseline values with measurements taken 7 days post-insertion.

**Results:**

The overall procedural success rate was 76.2% (16/21). The mean total insertion time was 40 ± 9.2 min, with a mean insertion depth of 101 ± 11.0 cm. The procedure demonstrated a favorable safety profile, with no significant perturbations in vital signs—heart rate, respiration, mean arterial pressure, or oxygen saturation—and no insertion-related complications were observed. Nutritional status improved significantly following intervention: serum total protein and albumin levels showed statistically significant improvement (*p* < 0.05), while prealbumin levels exhibited a strong trend toward improvement, approaching statistical significance (*p* = 0.05). In contrast, infection markers showed no statistically significant changes post-procedure (*p* > 0.05). Learning curve analysis indicated rapid skill acquisition among nursing staff, with procedural time stabilizing between 30–50 min after an initial learning phase.

**Conclusion:**

The pharmaco-mechanical synergy protocol for blind NET insertion in frail older adults with chronic wounds is associated with high success rates and a favorable safety profile. Our findings suggest this approach significantly improves nutritional status independent of underlying inflammatory states and demonstrates a replicable learning curve. Combined with its low-cost nature, this protocol represents a promising strategy for broad clinical dissemination, particularly in resource-conscious healthcare environments.

## 1 Introduction

Against the backdrop of a progressively aging society, issues within the field of geriatric medicine are garnering increasing attention. Frailty syndrome (FS) constitutes a major focus of active research in geriatrics and gerontology. Although its definitions vary, the most prevalent diagnostic criteria require the presence of three or more out of five phenotypic indicators: exhaustion, slowness, weakness, weight loss, and low physical activity. Frailty syndrome acts as an “invisible killer” lurking within the health of the older adult population, exerting a profound impact on physical function and quality of life (1). Malnutrition is both a cause and a consequence of frailty syndrome in older adults, forming a vicious cycle (2). Concurrently, chronic wounds are highly prevalent in this demographic. These wounds not only cause physical suffering but also significantly impair patients’ capacity for self-care and their psychological well-being (3). The complex interplay between these conditions presents a critical challenge in clinical nursing care for this specific patient population.

Relevant studies indicate that among older adults with chronic wounds, the prevalence of nutritional risk is 44.6, and 49.51% exhibit hypoalbuminemia (4), highlighting a concerning nutritional status that demands heightened clinical attention. Both international and national guidelines (5, 6) recommend post-pyloric enteral feeding via nasoenteric tube (NET) placement for such patients. This approach leverages the barrier function of the pylorus and the rapid emptying of the small intestine to reduce reflux risk. However, NET insertion is a technically complex and challenging procedure. Blind insertion at the bedside is associated with difficulties such as tube coiling and mucosal injury.

Current clinical practice also employs NET placement under endoscopic, fluoroscopic, or ultrasonic guidance (7, 8). While potentially improving accuracy, these methods necessitate specialized personnel and expensive equipment, and carry inherent procedural risks. These factors significantly limit their applicability for bedbound older adult patients. Nonetheless, promising bedside techniques have emerged. For instance, a repeated guidewire adjustment method for blind NET insertion has achieved an accuracy rate of 92.3% (9). Another approach involving postural modification combined with low-temperature catheter processing has been shown to optimize the esophago-gastric angle, enhancing the likelihood of successful tube passage (10). Based on the distinct pathophysiological challenges in frail older adults, this study developed and pilot-tested an integrated pharmaco-mechanical synergy protocol. This novel approach combines intravenous metoclopramide administration with a refined blind insertion technique—incorporating specific postural adjustments and a pulsed propulsion method—to address the technical difficulties and high risks associated with nasoenteric tube placement in this vulnerable population. The primary study objectives were to evaluate the protocol’s safety, feasibility, and preliminary effectiveness. The findings aim to establish a foundational framework and generate robust hypotheses for future definitive trials.

## 2 Subjects and methods

### 2.1 Study participants

We retrospectively analyzed data from older adult patients with chronic wounds who underwent bedside blind nasoenteric tube (NET) insertion in the Burn and Wound Repair Department between October 20, 2021, and May 16, 2025. Collected data included gender, age, primary diagnosis, indications for NET placement, and other observational metrics.

#### 2.1.1 Inclusion criteria

Patients were included if they met the following criteria:

1. Age ≥ 60 years;
2. Presence of a Stage IV unhealed wound documented in medical records;
3. Signed informed consent for NET placement obtained from the patient or legal guardian;
4. Meeting at least one of the following indications for NET use: intolerance to gastric feeding (e.g., gastric retention), high risk of reflux or aspiration (impaired consciousness, swallowing dysfunction), or failure to meet nutritional requirements despite oral intake.

#### 2.1.2 Exclusion criteria

1. Patients were excluded based on the following:
2. Severe nasal obstruction, pharyngeal edema, esophageal obstruction, pyloric stenosis, or contraindications for upper gastrointestinal intubation (e.g., post-esophagectomy or gastrectomy);
3. Recent gastrointestinal bleeding or high risk of bleeding (severe esophageal varices, significant coagulation disorders);
4. Contraindications for proximal enteral nutrition (e.g., distal intestinal obstruction or perforation);
5. Recent gastrointestinal surgery;
6. Terminal illness with palliative care goals;
7. QT interval prolongation > 450 ms.

### 2.2 NET insertion protocol

#### 2.2.1 Pre-insertion assessment and preparation

1. All patients underwent comprehensive evaluation prior to the procedure, including:
2. Frailty assessment using the Clinical Frailty Scale (CFS);
3. Swallowing function evaluated via the Standardized Water Swallow Test;
4. Nasopharyngeal anatomical structure assessment;
5. Coagulation profile.

Equipment prepared included a CH10 single-lumen spiral nasoenteric tube, metoclopramide injection (10 mg/vial), sterile saline, 5 mL and 20 mL syringes, enteral feeding sets, pH test strips, gauze, and nasal fixation devices.

#### 2.2.2 Pharmacological pre-treatment

Metoclopramide (10–20 mg) was administered intravenously 30 min prior to insertion to enhance antral contractions via dopamine D2 receptor antagonism (11). Contraindications including Parkinson’s disease, gastrointestinal bleeding, and QT interval prolongation > 450 ms were screened prior to administration.

#### 2.2.3 Patient preparation

Patients were kept fasting for at least 2 h pre-procedure. Oral and nasal secretions were suctioned to minimize aspiration risk during insertion.

#### 2.2.4 Two-operator collaborative technique

Given the cognitive impairment and poor cooperation common in frail older adults, a two-operator approach was adopted: a primary operator performed the insertion while an assistant maintained patient positioning, monitored vital signs, and provided reassurance.

#### 2.2.5 Modified insertion technique

A segmental approach was employed, optimized for different anatomical regions:

1. Catheter Pre-treatment: The NET was soaked in sterile saline for 20 min to activate its hydrophilic coating, enhancing flexibility and reducing mucosal trauma (12).
2. Esophageal Phase: With the patient in a semi-Fowler’s position, the tube was gently advanced through the nasal cavity. As it reached the pharynx, the assistant flexed the patient’s head forward toward the chest while lifting the jaw to close the glottis, preventing tracheal entry.
3. Gastro-pyloric Phase: The patient was rotated to a right lateral decubitus position. After instilling 50–100 mL of warm water, a pulsed advancement technique (2 cm forward, 0.5 cm back) was used, leveraging gravity to align the esophagogastric angle.
4. Post-pyloric Phase: Intermittent air insufflation (10–20 mL per 5 cm advancement) combined with pulsed propulsion facilitated pyloric passage by distending the gastric antrum.

#### 2.2.6 Tube tip confirmation

A multi-modal verification approach was employed:

1. Whoosh Test: Rapid air injection (20 mL) with simultaneous epigastric auscultation;
2. Vacuum Test: Aspiration of <20 mL air after 60 mL insufflation suggested transpyloric placement;
3. pH Testing: Gastric (pH ≤ 4) vs. intestinal (pH ≥ 7) aspirate differentiation;
4. Guidewire Recoil Test;
5. Abdominal X-ray (gold standard), confirming tip position ≥20 cm beyond the pylorus.

#### 2.2.7 Post-insertion management

Gastric decompression was maintained for 2 h post-confirmation of correct placement to reduce aerophagia.

### 2.3 Outcome measures

#### 2.3.1 Insertion success

Defined as radiographic confirmation of the tube tip distal to the pylorus.

#### 2.3.2 Procedural metrics

Included insertion time, tube depth, success rate, and complications (e.g., epistaxis, aspiration) or post-procedural events (occlusion, displacement).

### 2.4 Statistical analysis

Dual data entry and management were performed in Excel. Analyses used IBM SPSS 26.0. Continuous variables were tested for normality (Shapiro–Wilk, *α* = 0.05). Normally distributed data are presented as mean ± SD and compared using t-tests; categorical variables as frequencies (percentages) and compared via Fisher’s exact or Chi-square tests. Statistical significance was set at *p* < 0.05.

## 3 Results

### 3.1 Patient characteristics and procedural outcomes

A retrospective analysis was conducted on elderly patients with chronic wounds who underwent bedside blind nasoenteric tube insertion using the pharmaco-mechanical synergy protocol in our Burn and Wound Repair Department between October 20, 2021 and May 16, 2025. The cohort consisted of 14 males and 7 females, with a mean age of 75.2 ± 9.5 years. Clinical Frailty Scale (CFS) scores ranged from 7 to 9, indicating severe frailty (Table 1).

**Table 1:** Baseline characteristics of the study participants (*N* = 21).

Characteristic	Value
Patients, *n*	21
Gender, *n* (%)	
Male	14 (66.7)
Female	7 (33.3)
Age group, *n* (%)	
≥60 and < 65 years	4 (19.0)
≥65 and < 75 years	8 (38.1)
≥75 and < 85 years	4 (19.0)
≥85 years	5 (23.8)
Age (years)	75.2 ± 9.5
Clinical Frailty Scale (CFS) Score, *n* (%)	
7	19 (90.5)
9	2 (9.5)

The mean procedural time was 40 ± 9.2 min, with an average insertion depth of 101 ± 11.0 cm. The overall success rate was 76.2%. No procedure-related complications or unplanned extubations were recorded (Table 2).

**Table 2:** Nasoenteric tube insertion procedure outcomes (*N* = 21).

Procedural characteristic	Value
Insertion success, *n* (%)	16 (76.2)
Procedure time (min), Mean ± SD	40 ± 9.2
Insertion depth (cm), Mean ± SD	101 ± 11.0
Indication for insertion, *n* (%)	
Prolonged bed rest	11 (52.4)
Critical illness	7 (33.3)
Other	3 (14.3)
Tube tip position, *n* (%)	
Jejunum	14 (66.7)
Duodenum	2 (9.5)
Stomach	5 (23.8)
Procedure-related complications, *n* (%)	0 (0)
Unplanned extubation, *n* (%)	0 (0)

Vital signs remained stable throughout the procedure, with no statistically significant differences observed in heart rate, respiratory rate, mean arterial pressure, or oxygen saturation before and after tube placement (*p* > 0.05, Table 3).

**Table 3:** Comparison of vital signs before and after nasoenteric tube insertion (Mean ± SD).

Time	*n*	Heart rate (beats/min)	Respiratory rate (breaths/min)	Oxygen saturation (SpO₂, %)	Mean arterial pressure (mmHg)
During insertion	21	89.2 ± 13.3	18.9 ± 2.1	99.8 ± 0.8	87.4 ± 11.5
1 hour post-insertion	21	90.9 ± 14.1	19.4 ± 1.7	99.7 ± 0.6	88.7 ± 11.5
*p*-value		0.466	0.372	0.915	0.340

### 3.2 Nutritional parameters before and after tube insertion

The specific analysis of nutritional biomarkers at baseline (pre-insertion) and on day 7 post-procedure is shown in Tables 4, 5.

**Table 4:** Nutritional parameters before and after post-pyloric tube insertion (Mean ± SD).

Time	Serum total protein (g/L)	Serum albumin (g/L)	Serum prealbumin (mg/L)
1 day before insertion	68.0 ± 11.8	30.0 ± 3.9	143.0 ± 60.7
7 days after insertion	74.6 ± 9.0	35.0 ± 4.3	153.8 ± 63.6

**Table 5:** Paired-samples T-test analysis of nutritional parameters.

Paired variable	Mean ± SD	95% confidence interval	Test statistic	*P* value	
		Lower bound	Upper bound		
Serum total protein (g/L)	−6.59 ± 6.02	−9.80	−3.38	*t* (15) = − 4.377	0.001
Serum albumin (g/L)	−5.03 ± 4.37	−7.45	−2.61	*t* (14) = − 4.456	0.001
Serum prealbumin (mg/L)	−10.74 ± 11.59	−21.46	−0.02	*t* (6) = − 2.452	0.050

### 3.3 Inflammatory biomarkers before and after tube insertion

The specific analysis of inflammatory biomarkers at baseline (pre-insertion) and on day 7 post-procedure is shown in Tables 6, 7.

**Table 6:** Inflammatory biomarkers before and after post-pyloric tube insertion (Mean ± SD).

Time	Procalcitonin (PCT, ng/mL)	White blood cell count (WBC, ×10^9^/L)	C-reactive protein (CRP, mg/L)
1 day before insertion	9.1 ± 23.8	14.1 ± 9.6	58.2 ± 49.5
7 days after insertion	1.0 ± 1.1	10.9 ± 6.0	38.1 ± 64.5

**Table 7:** Paired-samples T-test analysis of inflammatory biomarkers.

Paired variable	Mean ± SD	95% confidence interval	Test statistic	*P* value	
		Lower bound	Upper bound		
Procalcitonin (PCT, ng/mL)	8.09 ± 23.43	−8.66	24.85	*t* (9) = 1.093	0.303
White blood cell count (WBC, ×10^9^/L)	3.10 ± 8.27	−1.67	7.88	*t* (13) =1.403	0.184
C-reactive protein (CRP, mg/L)	20.08 ± 66.79	−20.28	60.45	*t* (12) =1.084	0.300

The asterisk (*) denotes that no specific test statistic was calculated because the comparison was not statistically significant (*p* > 0.05).

### 3.4 Post-procedural enteral nutrition tolerance

Enteral nutrition tolerance outcomes were as follows: 16 patients (76.2%) demonstrated good tolerance without nausea, vomiting, or abdominal distension; 3 patients (14.3%) developed abdominal distension and pain; and 2 patients (9.5%) died due to underlying disease progression unrelated to the procedure.

### 3.5 Learning curve analysis for tube insertion

**Figure 1:**
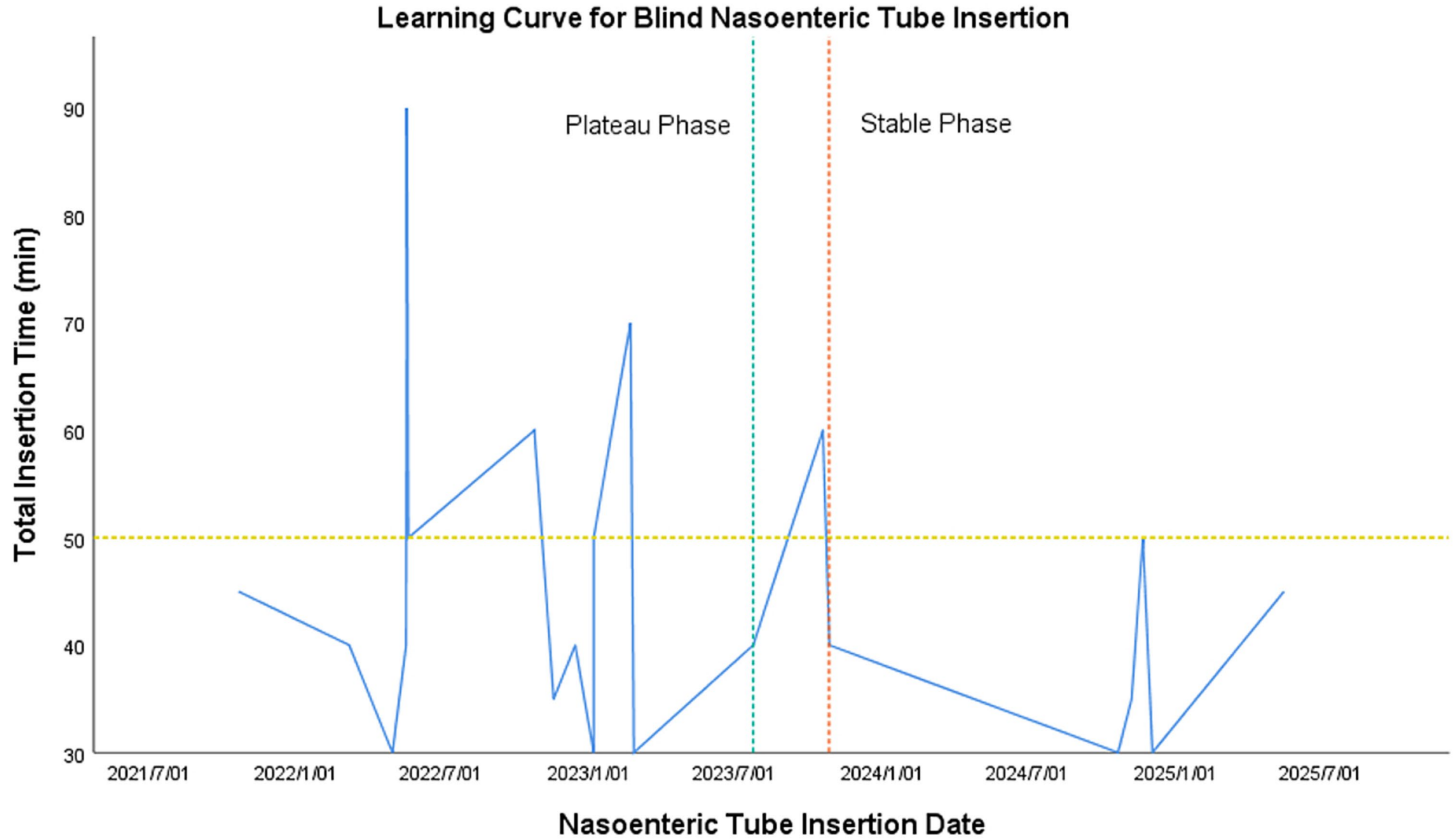
Learning curve for blind nasoenteric tube insertion.

A simple line graph was constructed with the procedural sequence by date on the x-axis and total insertion time on the y-axis, fitted with a trend line to evaluate the skill acquisition process and visually represent the change in operational efficiency as experience accumulated. By July 24, 2023, corresponding to the 14th procedure, the total insertion time was 40 min, indicating the operator had reached a plateau phase. Proficiency was achieved by the 16th procedure, demonstrated by notably reduced variation in operation time, which stabilized between 30–50 min (Figure 1).

## 4 Discussion

### 4.1 A systematic “pharmaco-mechanical synergy” approach as the core strategy for ensuring the safety and efficacy of tube placement in elderly frail patients

The patients enrolled in this study predominantly had a GPS score of 7–9, indicating a high degree of frailty characterized by depleted physiological reserves and poor cooperation, which places them at significantly elevated risk for complications during any clinical procedure (13). To address this challenge, our study implemented not a single technical modification, but an integrated “pharmaco-mechanical synergy” nursing protocol. This protocol systematically ensures procedural safety through three interlinked components. First, a comprehensive pre-procedural assessment identifies and excludes high-risk factors at the outset. Second, a dual-operator collaborative model effectively overcomes the uncertainties associated with poor patient cooperation and cognitive decline, ensuring stable positioning and real-time monitoring of vital signs. Most critically, the application of a segmented, modified insertion technique—including catheter pre-treatment with normal saline to reduce mucosal injury, targeted adjustments of head/body position, and a pulsed air injection advancement method—maximizes protection of the vulnerable nasopharyngeal and gastrointestinal anatomy.

This synergistic strategy of “pharmacological facilitation and mechanical guidance” represents the key advancement of our protocol over traditional direct insertion techniques. Pre-procedural intravenous administration of metoclopramide functionally enhances antral motility, creating a crucial “pharmacological time window” for the catheter tip to pass through the pylorus. Operational refinements tailored to the characteristics of elderly frail patients—such as ensuring an empty stomach, meticulous secretion aspiration, and the head tilt-chin lift maneuver—were implemented to address issues like poor cooperation, inadequate gastrointestinal motility, susceptibility to vomiting from gastric retention, and uncontrolled coughing or speaking. Concurrently, physical maneuvers like the right lateral recumbent position and pulsed air injection leverage gravitational and dynamic principles to guide precise catheter passage. The synergy between pharmacological and physical methods significantly increased the success rate of post-pyloric placement (14).

The absence of severe procedure-related complications, such as significant nasal mucosal injury or respiratory tract misplacement in all 21 cases, coupled with a successful placement rate of 76.2%, demonstrates the favorable safety and efficacy profile of this integrated protocol in this very high-risk population. This suggests that for elderly frail patients, the safety and effectiveness of nursing procedures stem from a systematic process grounded in risk assessment, meticulous attention to detail management, and multi-faceted synergistic safeguards.

### 4.2 The nutritional support protocol significantly improved patient nutritional status, independent of inflammatory state

As shown in Tables 4, 5, after 7 days of nasoenteral tube feeding, patients’ serum nutritional parameters showed significant improvement compared to pre-insertion levels. The serum total protein level increased from (68.0 ± 11.8) g/L pre-insertion to (74.6 ± 9.0) g/L post-intervention, with a mean increase of 6.59 g/L (95% CI: 3.38 to 9.80; *t* = −4.377, *p* < 0.001). Similarly, the serum albumin level rose from (30.0 ± 3.9) g/L to (35.0 ± 4.3) g/L, with a mean increase of 5.03 g/L (95% CI: 2.61 to 7.45; *t* = −4.456, *p* < 0.001). Prealbumin levels exhibited an increasing trend, with a mean rise of 10.74 mg/L (95% CI: 0.03 to 21.46; *t* = −2.452, *p* = 0.050). Given its short half-life (2–3 days), prealbumin is a more sensitive nutritional marker than albumin. This upward trend further corroborates the conclusion of improved nutritional status. It is noteworthy, however, that the significance was borderline, and the small sample size for this specific marker (*n* = 6) may have resulted in insufficient statistical power; thus, this finding requires further validation in larger future studies.

According to Tables 6, 7, regarding inflammatory markers, no statistically significant differences were observed between pre-insertion and day 7 post-insertion levels for procalcitonin, white blood cell count, or C-reactive protein (all *p* > 0.05). This indicates that the tube placement and feeding protocol did not alter the patients’ existing inflammatory state. Consequently, the improvement in nutritional status occurred independently of infection control and was not secondary to a resolution of inflammation. For frail elderly patients, who often present with a “chronic inflammatory state,” this finding underscores the pivotal role of proactive and aggressive nutritional support in reversing negative nitrogen balance and promoting anabolism, highlighting its independent importance. This conclusion aligns with findings from studies such as that by Tatsuro Inoue et al. (15).

### 4.3 The pharmaco-mechanical synergy protocol demonstrates significant clinical scalability based on learning curve and cost analysis

The learning curve presented in Figure 1 clearly demonstrates the acquirability and stability of this nursing technique. The post-pyloric tube placement procedure was established as a core nursing skill within our unit. Each procedure was performed by a different nurse, all under the direct supervision and guidance of a certified nurse manager experienced in the technique. A significant reduction in procedure time was observed, decreasing from an initial 60–90 min to a stabilized plateau of 30–50 min after approximately 14–16 cumulative procedures. This quantitative evidence indicates that the technique can be effectively mastered by nursing staff through standardized training, with a controllable and well-defined learning cost.

The direct costs associated with this procedure include CNY 372.2 for the enteral feeding tube and CNY 83 for a bedside X-ray, totaling CNY 455.2. From a health economics perspective, this cost is substantially lower than that of conventional techniques such as visualized placement (e.g., endoscopic or fluoroscopic guidance). This “low-cost, high-value” profile aligns not only with the core policy directives of China’s “Strengthening Primary Healthcare Capacity Initiative,” which emphasizes enhanced resource utilization and cost-effectiveness at the primary care level (16), but also with global health priorities. The World Health Organization (WHO) strongly advocates for Universal Health Coverage and promotes affordable, essential health technologies to reduce the global disease burden, particularly among the aging population (17). This protocol therefore represents a highly relevant and readily implementable strategy for low- and middle-income countries grappling with the dual challenges of rapidly aging populations and constrained healthcare budgets (18). It provides a practical, high-quality solution to overcome the technical bottlenecks in providing nutritional support for critically ill and frail patients in primary care settings—a solution that is nurse-performable, institution-affordable, and patient-beneficial—indicating considerable potential for widespread adoption.

## 5 Conclusion

This study provides preliminary evidence that the “pharmaco-mechanical synergy” nasoenteral tube placement protocol, conceptualized from a frailty syndrome perspective, demonstrates a favorable safety profile (with an absence of severe complications) and clear efficacy (evidenced by significant improvements in nutritional biomarkers such as serum albumin and total protein) in elderly frail patients. More importantly, the improvement in nutritional status occurred independently of changes in inflammatory markers, underscoring the central therapeutic role of proactive and aggressive nutritional support in this population.

The learning curve analysis further established the technique’s acquirability and stability, quantifying the training requirement at approximately 14–16 procedures and providing an empirical basis for its clinical dissemination. Combined with its low-cost profile, the protocol aligns well with national and international policy directives aimed at strengthening primary healthcare capacity, highlighting its potential as an appropriate technology for widespread adoption in community and resource-limited settings.

The primary limitation of this study is its exploratory nature, being a single-center investigation with a limited sample size (*n* = 21), which constrains statistical power and generalizability. However, the in-depth analysis of this cohort successfully revealed underlying patient heterogeneity, delineated the skill acquisition pattern, and generated valuable hypotheses. Consequently, the core contribution of this work lies in establishing a clear framework and direction for future large-scale, multi-center prospective studies. It lays a solid preliminary foundation for implementing “precision” nutritional support nursing in the geriatric frail population and provides well-defined targets for subsequent research and training protocols.

## Data Availability

The original contributions presented in the study are included in the article/supplementary material, further inquiries can be directed to the corresponding author/s.
